# A 69-year-old male patient presenting with chest pain and shortness of breath

**DOI:** 10.1007/s12471-019-1260-3

**Published:** 2019-03-12

**Authors:** Y. Saleh, K. Herzallah, M. Elsayed, S. Elkinany, S. Rayamajhi

**Affiliations:** Michigan State University Clinical Center, East Lansing, MI USA

## Answer

**Fig. 1:**
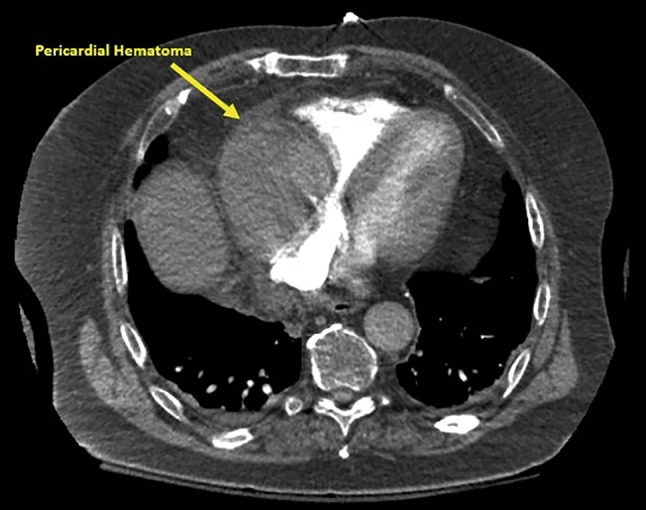
Computed tomography finding

Computed tomography demonstrated a solid soft-tissue density in the right aspect of the pericardium, significantly compressing the right atrium, measuring 9.1 × 7.4 cm in diameter (Fig. 1). Transthoracic echocardiography showed a good systolic function, a well-functioning aortic valve prothesis and a mass compressing the right atrium (Video 1). The patient underwent transoesophageal echocardiography-guided video-assisted thoracoscopic surgery and the pathology revealed that it was a localised haematoma. The haematoma was successfully drained, and the patient’s symptoms resolved afterwards (Video 2).

Haemopericardium secondary to warfarin can occur in patients presenting with pericarditis, coagulopathy or trauma. However, it is extremely rare to occur spontaneously. Usually it presents as circumferential effusion, unlike our patient who presented with a localised haematoma [1]. Haemopericardium is a well-known complication in the early postoperative phase after heart surgery, but in our patient it presented 35 years after aortic valve replacement.

## Caption Electronic Supplementary Material

​Transthoracic echocardiogram in modified parasternal short axis view at the level of the great vessels

​Transesophageal echocardiogram showing a mass compressing the right atrium
